# Active versus Passive Training of a Complex Bimanual Task: Is Prescriptive Proprioceptive Information Sufficient for Inducing Motor Learning?

**DOI:** 10.1371/journal.pone.0037687

**Published:** 2012-05-23

**Authors:** Iseult A. M. Beets, Marc Macé, Raf L. J. Meesen, Koen Cuypers, Oron Levin, Stephan P. Swinnen

**Affiliations:** 1 Motor Control Laboratory, Department of Biomedical Kinesiology, Katholieke Universiteit Leuven, Heverlee, Belgium; 2 Institut de Recherche en Informatique de Toulouse, Université Paul Sabatier, Toulouse, France; 3 REVAL - Rehabilitation and Health Care Research Center, Department of Health Care, University College of Limburg, Hasselt, Belgium; 4 Biomedical Research Institute, School of Life Sciences, Hasselt University Agoralaan 1, Diepenbeek, Belgium; National University of Singapore, Singapore

## Abstract

Perceptual processes play an important role in motor learning. While it is evident that visual information greatly contributes to learning new movements, much less is known about provision of prescriptive proprioceptive information. Here, we investigated whether passive (proprioceptively-based) movement training was comparable to active training for learning a new bimanual task. Three groups practiced a bimanual coordination pattern with a 1∶2 frequency ratio and a 90° phase offset between both wrists with Lissajous feedback over the course of four days: 1) passive training; 2) active training; 3) no training (control). Retention findings revealed that passive as compared to active training resulted in equally successful acquisition of the frequency ratio but active training was more effective for acquisition of the new relative phasing between the limbs in the presence of augmented visual feedback. However, when this feedback was removed, performance of the new relative phase deteriorated in both groups whereas the frequency ratio was better preserved. The superiority of active over passive training in the presence of augmented feedback is hypothesized to result from active involvement in processes of error detection/correction and planning.

## Introduction

Learning new motor skills is associated with a transition from a cognitive (attention-demanding) stage to an autonomous stage in which skill automaticity is accomplished [1]. Practice is critical for improving performance but also contextual elements, such as organization of practice, play an important role. Active involvement of the learner in processes of error detection and correction is critical for memory formation and retention. Moreover, depending on the complexity of the skill, external support from a trainer or therapist is invaluable for shaping skilled performance. This external support for learning consists of at least two modes. On the one hand, information can be provided about how the movement should be performed (prescriptive information), such as verbal instructions or demonstrations of the intended skill. On the other hand, external feedback that is complementary to the naturally available sources of information can be provided during or after completion of the trial (also called augmented feedback) [2], [3]. For learning to occur, it is critical that information about actual performance (what was done) can be compared to a standard referring to correct movement (what should be done). If the learner fails to conduct this comparison process, external help becomes more valuable.

With respect to prescriptive information, it has become clear that observation of action by means of a demonstration plays a critical role in learning and its neurophysiological substrate has been investigated in detail during the past years. More specifically, in the primate brain, mirror neurons have been discovered that are similarly recruited during observation of an action as during actual performance of that same action [4]. Transcranial magnetic stimulation (TMS) research in humans has revealed that observation of actions results in increased excitability in the primary motor cortex which receives input from the mirror neuron system [5], [6], [7], [8]. Indeed, the mere observation of actions can yield motor learning [9], [10]. This enhancing effect of visual perception on action is called ‘perception-to-action transfer’ and is a well-studied phenomenon [9] which underlines the importance of perceptual processes for supporting motor learning. In other words, motor learning is accompanied with perceptual learning [11].

Instead of prescriptive information, (augmented) visual or auditory feedback (FB) can be provided to the learner to assist skill acquisition. A variety of feedback types exist, ranging from verbal information about the general outcome of the action (knowledge of results: KR) to detailed information about movement kinematics or kinetics (knowledge of performance) [12], [13]. For example, in the context of learning interlimb coordination skills, use has been made of Lissajous figures, displaying the motion of 2 limbs or limb segments orthogonally to each other [2], [3], [14], [15], [16], [17], [18], [19]. This visualizes the quality of coordination directly by integrating the two separate signals into a meaningful gestalt. Feedback information can also be provided together with prescriptive information to direct the learner towards correct performance [20]. However, there is also a disadvantage to provision of augmented feedback because performance levels obtained in the presence of augmented feedback cannot always be maintained during future retention performance in the absence of augmented feedback [19], [21], [22].

Compared to visual input, there is relatively little evidence on the effect of provision of proprioceptive information for learning. This is understandable because it is less straightforward to provide prescriptive information or augmented feedback that targets the proprioceptive senses directly. Proprioception refers to the sense of the position and movement of the body (parts). The muscle spindles which encode information on muscle length and its rate of change (i.e., velocity) are considered major contributors to proprioception [23], [24], [25]. In training or rehabilitation settings, performers/patients are sometimes guided through a movement path to acquaint them with kinesthetic information associated with the correct movement. The limited experimental efforts on the impact of proprioceptive prescriptive information for learning are surprising because it is generally agreed that this is a critical source of input for motor performance and learning. Deafferented patients for example, are impaired in controlling reaching movements, coordination and force production [26], [27].

Studies comparing passive (feeling the movement) with active movement (doing the movement) are also instrumental in revealing the role of proprioceptive information for learning. This essentially refers to prescriptive information about correct movement experienced passively (rather than augmented feedback about the performer's own movement) which can be accomplished by means of torque motors and other devices. Recent research has shown that delivering proprioceptive prescriptive information of circular hand movement trajectories with an elliptical velocity pattern (passive practice) leads to a significant improvement in the active reproduction of this new motor skill [28], [29] and robots can assist in learning other motor tasks, such as steering a simulated vehicle [30]. Providing proprioceptive information may help build a template of expected sensory consequences [31] or forward models (e.g., [32], [33], [34]), that accompany the acquisition of the skill. Prescriptive proprioceptive information may support the development of a new sensory representation of the goal movement which is retrieved during movement production as predicted by the classic motor theory of Schmidt [31] and Adams [35], [36]. Whether proprioceptive information provision influences learning of a new bimanual coordination pattern has hardly been determined.

To investigate the effect of passive as compared to active movement in more detail, we made use of a complex bimanual movement that required participants to acquire a new spatiotemporal organization between the limbs [37]. Participants were trained to perform a complex coordination task in which both hands executed a 1∶2 frequency ratio (L∶R = 1∶2) with a 90° phase offset over the course of four days (for a similar forearm task paradigm, see [19]. This allowed us to trace the acquisition of the frequency ratio (global inter-cycle timing) as well as the relative phasing between the limbs (spatiotemporal coordination, or intra-cycle timing). Executing a new frequency ratio and a new relative phasing simultaneously may be more complex than either of these separately because frequency as well as phase constraints have to be overcome. Nevertheless, both measures are neither totally independent nor dependent because a new unified pattern emerges from the combination of both (see also task description). Here, we investigated whether provision of prescriptive proprioceptive information about correct performance supported acquisition of both task features. Therefore, performance of a passive training group was compared with an active training group and a control group not involved in practice. To the best of our knowledge, this is the first time in the literature that these practice groups are addressed in the context of new coordination learning.

Based on the observation that active and passive movement experience generates activation in partially similar neural substrates [38], [39], [40], we hypothesized that passive training (prescriptive proprioceptive information) would induce improvement with practice. More specifically, we hypothesized that the passive and active training groups would perform better than the control group and that the active training group would outperform the passive group because the former would benefit from error detection and correction processes associated with self-produced movements. Functional magnetic resonance imaging (fMRI) work for example, has shown that active as compared to passive training led to more prominent increases in activation of contralateral primary motor cortex (cM1) during retention tests and higher corticospinal facilitatory effects (as measured by TMS induced motor evoked potentials) [41]. With respect to frequency ratio versus relative phase learning, we anticipated that the former would be easier to acquire and retain than the latter because humans are hypothesized to be generally more acquainted with macroscopic timing (i.e., between cycles) than with microscopic spatiotemporal movement organization (within cycles) throughout development, particularly when integer frequency ratios are performed [42], [43], [44]. Nevertheless, acquiring these different features will also depend on the availability of perceptual information to promote perception-action integration and the ability to perceive the given coordination pattern [37], [45], [46], [47].

## Materials and Methods

### Participants

Thirty volunteers (18 male, 12 female; mean age 21.1 yrs, range: 18–29) without any known neuromuscular disorders participated in this study. All participants were right-handed, as assessed with the Edinburgh Handedness Questionnaire [48], were naive about the purpose of the experiment and had no previous experience with the task. Written informed consent was obtained before the experiment, and the experimental procedure (in accordance with the Declaration of Helsinki) was approved by the local Ethics Committee for Biomedical Research at the Katholieke Universiteit Leuven.

### Apparatus and task

**Figure 1:**
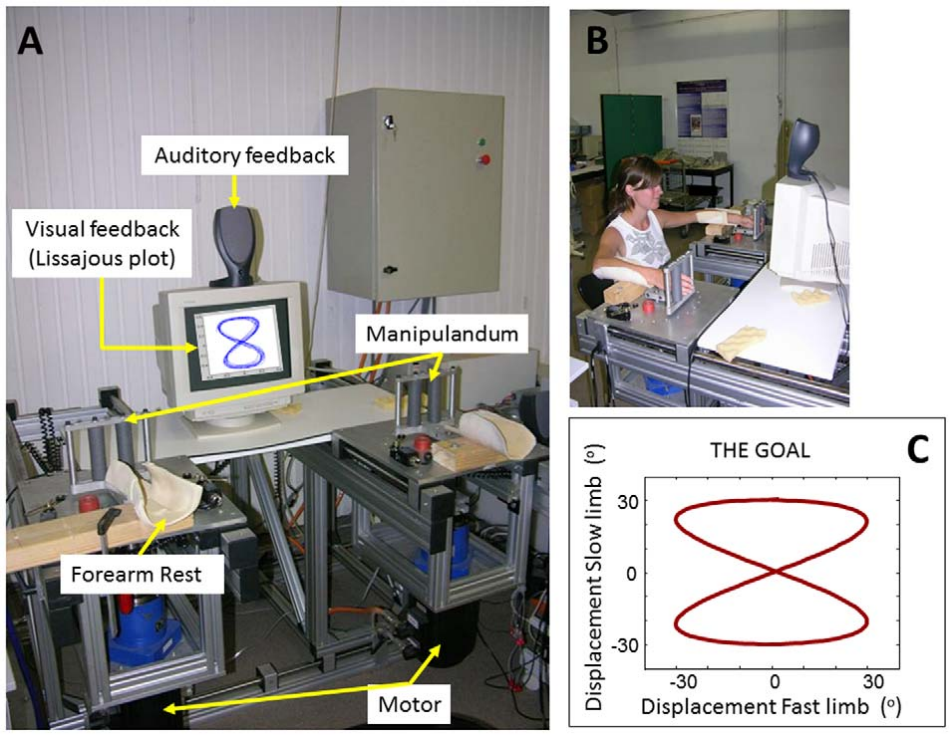
Experimental setup. A. Apparatus consisting of torque motors, forearm rests, moving hand pieces, and a pc providing feedback. B. Positioning of participants during task execution. C. Lissajous plot of the goal (1∶2 frequency ratio with a 90° phase offset between limbs) produced by both limb displacements.

A purpose-built apparatus (Figure 1A) was used to impose flexion-extension movements to the wrist. The apparatus consisted of two separate units (left and right), both fitted with a forearm rest to support it in a natural position and a manipulandum for insertion of the hand palm. Motion of the wrists was induced by means of AC servo motors (AMK DV764, Goedhard PMC, Helmond, the Netherlands) that were mounted underneath each unit and coupled to the rotating shaft of the manipulandum via a 1∶10 reducer (Alpha Gearbox, Type LP120) and mechanical clutches. The motor generated a continuous sinusoidal motion of a programmable amplitude, frequency and duration, allowing rotation of the wrist from −30° (flexion) to +30° (extension), relative to a 0° position (whereby the forearm and the palmar surface of the hand were aligned). Analog potentiometers were connected to the rotating axes (accuracy = 0.088°) to record the angular displacement signals. Movement signals were sampled at 200 Hz (Power 1404 CED device; Cambridge Electronic design, Cambridge, UK). Online visual feedback of the displacement-displacement angles of the two wrists was provided on a PC screen that was positioned in front of the subject. The motions were plotted orthogonally with the left limb movement represented on the ordinate and the right limb movement on the abscissa (Lissajous plot). To pace the frequency of the 1∶2 movements, auditory signals were provided with a loudspeaker placed on top of the PC screen, in front of the subject.

Participants were seated on a height-adjustable chair in front of the apparatus such that the body was aligned between the lever axes with their shoulders positioned in slight abduction (10–20°), elbows at 90° and forearms supported in a neutral pro-supination position (Figure 1B). Participants were instructed to make cyclical, bimanual wrist movements with a 1∶2 frequency ratio and with a 90° phase offset between the limbs. Movements of the fast (right) limb were paced by an electronic metronome producing a beep every 750 ms (1.33 Hz) whereby every second beep was stressed to pace the slower (left) limb movement (0.66 Hz). For the active movements, 1000 Hz and 1400 Hz auditory signals were given (80 ms), which were associated with the left and right limb movement, respectively. Onsets of the tones (during passive movement) were synchronized with the motion of the two torque motors. Subjects were required to complete an entire movement cycle with their fast moving limb on every beat, while the slow moving limb only performed a flexion or extension motion cycle for every stressed beat. As such, the fast limb reached the same turning point on every beep, whereas the slow limb reached the same turning point on every stressed beep. The same metronome beats were also used during passive training.

**Figure 2:**
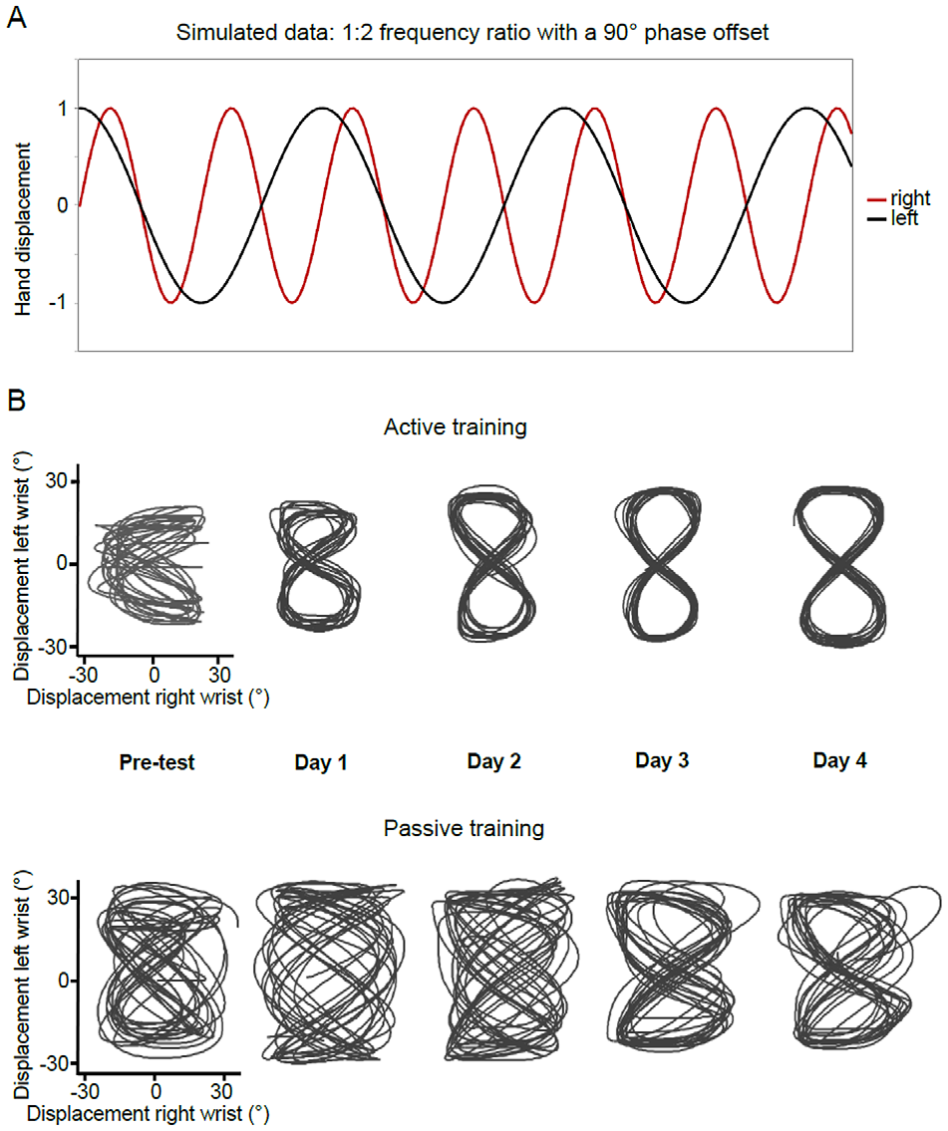
Movement goal and reproduction. A. Visual representation of the target coordination pattern according to the required 1∶2 frequency ratio with a 90° phase offset. The relative phase was calculated at the turning points, where the goal was 90°. B. Lissajous plots of active movement production in test trials where feedback was present for a representative subject in the active (top) and the passive (bottom) training groups during pretest and across training days.

For both wrists, the required movement amplitude was 60° peak-to-peak, indicated by the boundaries of the target Lissajous-plot. When produced correctly, the task resulted in a Lissajous plot with a figure-of-eight configuration (Figure 1C, movement kinematics shown in Figure 2A). This figure illustrates a relative motion plot of two pure sine waves with equal amplitudes, a 1∶2 frequency ratio, and with a phase offset of 90° (for a similar task, see [19]).

### Procedure

Subjects were randomly divided into three experimental groups. The first group (N = 10; 7 male, mean age: 20.8±1.8 years) practiced the required coordination task (1∶2 frequency ratio movement with 90° out of phase) actively (Active Training Group) whereas the second group (N = 10; 7 male, mean age: 21.8±2.8 years) acquired the task with passive training via the movements of the torque motors (Passive Training Group). The third group (N = 10; four male; mean age = 20.6±2.1 years) did not practice the task (Control). In both training groups, movements were practiced across 4 consecutive days, i.e., 4 sessions per day each consisting of 25 practice trials, with a duration of 20 s per trial. Following each practice session, participants received a 5 min rest interval. Four test trials were performed prior to and after completion of practice on each day. These test trials consisted of two trials (20 s/trial) in which the movement was performed actively in the absence of on-line visual and auditory information followed by two trials in which participants received visual feedback and auditory pacing (Table 1). The no-feedback test trials were included to assess the degree of preservation of the acquired coordination patterns in the absence of concurrent information feedback. The control group did not undergo training, but performed the same test trials over four consecutive days, similar to the other two groups. More specifically, each day consisted of two sessions of 4×20 s test trials that were separated by a 50 min rest period, instead of training (Table 1). This group was included to assess practice effects caused by performing the test trials only. Finally, all three groups completed a retention test consisting of four active test trials i.e., 2 trials without augmented visual feedback and auditory pacing and 2 with visual feedback and auditory pacing one week after the end of the last practice day.

**Table 1:** Experimental protocol for the three groups, indicating the timing/number of performance tests (Pre/Post), feedback conditions (−: no concurrent feedback/+: with concurrent feedback), practice days, practice session and number of blocks×practice trials/session.

	Active Group and Passive Group					Control Group				
	Day 1	Day 2	Day 3	Day 4	Retention	Day 1	Day 2	Day 3	Day 4	Retention
Pre-test	4 −/+	4 −/+	4 −/+	4 −/+	4 −/+	4 −/+	4 −/+	4 −/+	4 −/+	
Session 1	25 +	25 +	25 +	25 +						
Session 2	25 +	25 +	25 +	25 +						
Session 3	25 +	25 +	25 +	25 +						
Session 4	25 +	25 +	25 +	25 +						
Post-test	4 −/+	4 −/+	4 −/+	4 −/+		4 −/+	4 −/+	4 −/+	4 −/+	

Each performance test consisted of four active movement trials (duration = 20 s): two without augmented feedback (−) followed by two trials with feedback (+).

Participants in the passive training group were instructed to keep their wrist muscles relaxed at all times and not to resist/assist the motion induced by the torque motors during the passive training trials. To test whether participants complied with those instructions, EMG activity of the flexor (FCR) and extensor (ECR) carpi radialis of the right and left wrists was recorded. Signals were collected by means of disposable, Ag-AgCl, surface electrodes (Blue Sensor SP) that were placed over the middle portion of the muscle belly, and aligned with the longitudinal axis of the muscles. EMG signals were amplified (×1000, MEGA MSPEC 8000), bandpass filtered (4–500 Hz), sampled at 1000 Hz (Power 1404 CED device) in parallel to the motion signals and were monitored on-line by the experimenter. We did not analyze the EMG data further, as visual inspection verified that muscles were relaxed and did not show activation patterns resembling those during active movement production.

### Data analysis

The data analysis focused on the evolution of the accuracy and consistency of the 1∶2 frequency ratio and relative phasing as a function of practice. Relative phase and cycle duration of the displacement signals were calculated for each motion cycle. Relative phase was defined as the subtraction of the phase angle of the left (slow) from the right (fast) wrist according to Kelso et al. (1986):
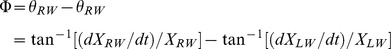
where 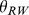 refers to the phase of the right wrist movement, 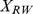 is the position of the right wrist after rescaling to the interval [−1,1] for each cycle of oscillation, and 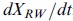
is the normalized instantaneous velocity. Following computation of the continuous estimate of relative phase with the formula shown above, the absolute difference in phase angle (ranging from 0° to 180°) was extracted at two peak position landmarks (i.e., the turning points or direction reversals) of the reference (right) limb and for each oscillation cycle. Note that the target relative phase was 90° at these turning points, but not at other epochs during the cycle because relative phase wraps around as a result of the 1∶2 frequency relation (see Figure 2A). Subsequently, measures of coordination accuracy and consistency were determined. The mean absolute error of relative phase (AEΦ) reflected the absolute deviation from the target relative phase, i.e., 90° (‘coordination accuracy’). The standard deviation of relative phase (SDΦ) referred to the spread of relative phase measures around the mean (‘coordination consistency’).

Cycle duration was defined as the time that elapsed between successive peak extension positions. The average cycling frequency of the right (CycFR) and left (CycFL) wrist movements were computed for each oscillation cycle. Subsequently, the cycling frequency ratio (CFR) between the right (fast) and left (slow) limbs was calculated: CFR = CycFR/CycFL. This parameter provides a direct quantification of how well participants comply with the 1∶2 frequency ratio. The absolute deviation from the required CFR = 2 (AE CFR) was computed across the 20 s trial to assess temporal accuracy. Within-trial standard-deviation of the cycling frequency ratio (SD CFR) was computed to assess temporal variability.

The dependent variables AEΦ, SDΦ, AE CFR and SD CFR were analyzed by means of a 3×5×2 analysis of variance (ANOVA) with the factors Group (3 levels: Active, Passive, Control), Day (5 levels: Day 1 Pre-test and Day1, Day2, Day 3, Day 4 Post-test) and Feedback (2 levels: presence vs. absence of concurrent visual feedback) and with repeated measures on the last two factors. Results are summarized in Table 2. Furthermore, we investigated the outcome of training groups by analyzing performance during retention with a 3×2 (Group×Feedback) ANOVA. Finally, we directly tested the change in performance between pre-test and retention to determine learning effects. To this end, we calculated the improvement percentage by the formula: −((Perf _retention_−Perf _pre-test_)/Perf _pre-test_)×100. ‘Perf’ is the obtained AE or SD per participant. Note that the sign was inversed to transform the error reduction into a percentage of improvement. 3×2 ANOVAs were conducted with factors Group×Type of Metric (relative phase, cycling frequency ratio). For all analyses, the probability level was set at *p*<0.05, 2-sided. When significant effects were found, post-hoc tests (Tukey HSD, which corrects for multiple comparisons) were conducted to identify the loci of these effects.

**Table 2:** Results of the 3×5×2 ANOVAs (*F*-values) for absolute error (AE) and variability scores of relative phase (SDΦ) and cycling frequency ratio (CFR).

	Df	Relative Phase (Φ)		Cycling Frequency Ratio (CFR)	
		AE (Φ)	SD (Φ)	AE (CFR)	SD (CFR)
Group	2,27	24.5***	6.51**	10.8***	9.56**
Feedback	1,27	53.7***	15.0**	3.96†	0.73
Day	4,108	13.1***	2.08†	22.9***	1.53
Group×Feedback	2,27	13.0***	10.6***	1.20	1.96
Group×Day	8,108	4.14***	5.0***	0.27	1.24
Day×Feedback	4,108	4.36**	4.63**	2.23†	1.23
Group×Day×Feedback	8,108	7.35***	1.56	1.46	0.72

* *p*<0.05,

** *p*<0.01,

*** , *p*<0.001.

† Marginally significant (*p*<0.1).

## Results

### General learning effects

Representative examples of relative motion (Lissajous) plots obtained at pre-test (Day 1) and at the end of each day of practice (Day 1 to Day 4) in the presence of augmented feedback are presented in Figure 2B for participants in the active (top) and passive (bottom) training groups. The displacement signals of the non-dominant (slow) and dominant (fast) limbs are plotted on the ordinate and abscissa, respectively. At pre-test, the relative motion plots were highly inconsistent, indicating that participants in both groups were not able to produce the required coordination pattern before initiation of practice (left hand panel, Figure 2). By the end of the first day of practice, participants in the active training group were able to produce the 1∶2 frequency ratio more correctly with a 90° phase offset between the limbs, as can be seen in the second panel of figure 2A showing the ‘figure 8’ configuration. From the second day of practice, the variation in the 8-shaped trajectory across cycles further declined, reflecting increased performance consistency. Finally, performance was nearly perfect toward the end of practice (top right hand panel, Figure 2B). In comparison with the considerable progress being made during active training, more difficulties were experienced during passive training (bottom panels, Figure 2B). By the end of the third and fourth day of practice, the figure-8 configuration became apparent but it could not be maintained consistently throughout the trial. These observations are further discussed in relation to the group results, using separate 3×5×2 (Group×Day×Feedback) ANOVAs on each of the performance scores (see Table 2).

#### Performance of the bimanual movement pattern: phase relation

**Figure 3:**
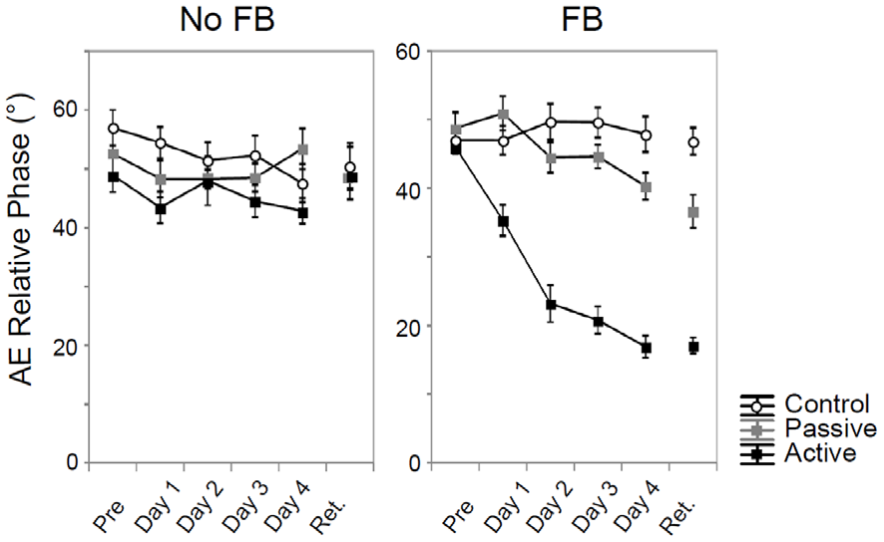
Performance of absolute error of relative phase across days. Absolute error (AE) of the required relative phase (90) in degrees (°) across practice for each group in the absence (left) and presence of augmented feedback in the form of a Lissajous plot (right). Error bars represent SE of mean.

Accuracy. Group data of the mean relative phase error (AEΦ) are shown for trials in the absence and presence of augmented visual feedback (Figure 3). Performance error was high across all three groups when augmented feedback was not available and it remained high across days (see Figure 3, left). However, during trials with augmented feedback, the active group showed a decrease in error across practice days as compared to the passive group who showed less improvement (see Figure 3, right), while no improvement was observed for the control group. This observation is supported by the significant Group×Day×Feedback interaction (see Table 2).

Additional Group×Day ANOVA's were conducted for each feedback condition separately. In the absence of augmented feedback, there were neither significant effects for Group [*F*(2, 27) = 2.72; *p* = 0.084] (active: *M* = 45.53; passive: *M* = 50.22; control: *M* = 52.54) and Day [*F*(4, 108) = 2.06; *p* = 0.091], nor was the Group×Day interaction [*F*(8, 108) = 1.35; *p* = 0.23]. In the presence of augmented feedback however, there was a significant Group×Day interaction [*F*(8, 108) = 12.06; *p*≤0.00001]. The difference between the active vs. both the control and the passive group became significant from Day 1 on [all *p*<0.001]. There were significant main effects of Group [*F*(2, 27) = 74.94; *p*≤0.00001] (active: *M* = 28.44; passive: *M* = 45.79; control: *M* = 48.21) and Day [*F*(4, 108) = 19.47; *p*≤0.00001].

**Figure 4:**
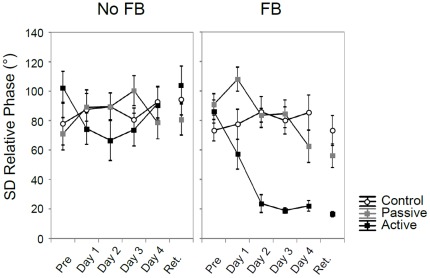
Performance of standard deviation of relative phase across days. Standard deviation (SD) of relative phase in degrees (°) across practice for each group in the absence (left) and presence of augmented feedback in the form of a Lissajous plot (right). Error bars represent SE of mean.

Variability. The general picture for SDΦ looked similar to that of AEΦ (see Figure 4 & Table 2). There were significant main effects for Group and Feedback. The three-way Group×Day×Feedback interaction did not reach significance, whereas significant lower order Group×Feedback, Group×Day, and Day×Feedback interactions were observed. The Day×Feedback interaction indicates that the decrease in variability scores across days was present only in the presence of augmented feedback (Figure 4). The significant Group×Feedback interaction suggested that no differences among the three groups were observed in the absence of augmented feedback. However, when feedback was present, the active training group showed a sharp drop in SDΦ scores as compared to the passive and control groups (active: *M*_NFB_ = 81.83, *M*_FB_ = 41.61; passive: *M*_NFB_ = 85.65, *M*_FB_ = 85.89; control: *M*_NFB_ = 85.65, *M*_NFB_ = 80.36) [all *p*<0.001]. The Group×Day interaction suggests that only a clear improvement in SDΦ (Feedback conditions collapsed) across days was evident in the active training group. Participants in the control and the passive training group experienced difficulties in stabilizing their movements across days.

To summarize, coordination accuracy and consistency of relative phase improved significantly in the active training group, but only in the presence of concurrent feedback.

#### Performance of the bimanual movement pattern: 1∶2 frequency ratio

**Figure 5:**
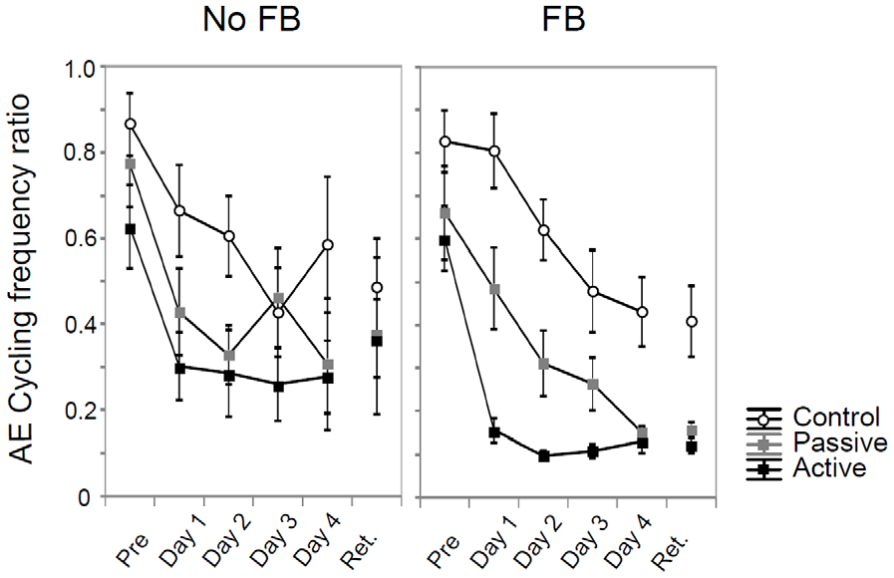
Performance of frequency ratio across days. Frequency ratio error changes across practice for each group in the absence (left) and presence of augmented feedback in the form of a Lissajous plot (right). Error bars represent SE of mean.

Accuracy. The 3×5×2 Group×Day×Feedback ANOVA (see Figure 5 & Table 2) revealed significant main effects for Group (active: *M* = 0.28; passive: *M* = 0.42; control: *M* = 0.63) and Day. Over days, a significant reduction in error was seen (in order, from pre-test to Day 4: *M* = 0.73; 0.47; 0.37; 0.33; 0.31). The active training group again outperformed the control group [*p* = 0.00036], but not the passive group [*p* = 0.20]. The passive group was even superior to the control group [*p* = 0.022]. None of the interaction effects reached significance.

Variability. The ANOVA (see Table 2, figure is not shown) only revealed a significant main effect of Group (active: *M* = 0.20; passive: *M* = 0.23; control: *M* = 0.33). Post-hoc tests demonstrated that the active group performed the frequency ratio with lower variability than the control group [*p* = 0.00099] but performance did not differ significantly from the passive group [*p* = 0.69]. The passive group also outperformed the control group [*p* = 0.0074]. None of the remaining main and interaction effects were significant (see Table 2).

In summary, whereas the passive group showed difficulties in acquiring the relative phase pattern relative to the active group, they showed comparable improvement in both accuracy and consistency of the 1∶2 frequency ratio.

### Retention of performance as an indication of training outcome

**Figure 6:**
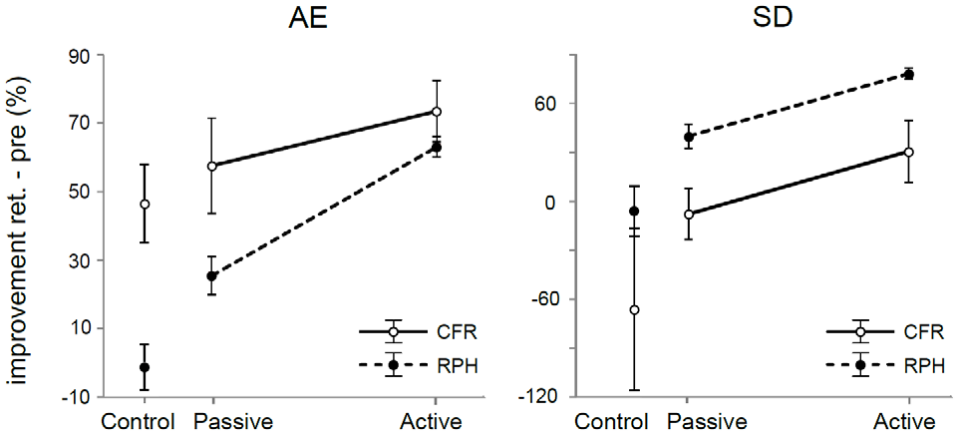
Improvement from Pre-test to Retention. Improvement (in percentage), i.e., inverse of error decrease, of Retention compared to Pre-test under feedback per group for AE (left) and SD (right). Filled dots & striped lines: improvements on relative phase (RPH); Open dots & solid lines: improvements on relative cycling frequency ratio (CFR).

Retention was tested one week following the final training day. In addition, we analyzed the percentage of improvement between pre-test and retention which will be compared directly across performance measures (see Figure 6).

#### Performance of the bimanual movement pattern: phase relation

Accuracy. The 3×2 (Group×Feedback) ANOVA on retention scores revealed a significant main effect for Feedback [*F*(1, 24) = 46.29; *p*≤0.00001] and Group [*F*(2, 24) = 10.97; *p* = 0.00041], as well as a significant Group×Feedback interaction [*F*(2, 24) = 12.68; *p* = 0.00017] (see Figure 3). Separate ANOVAs were therefore conducted per feedback condition. While performance was similar among groups under nonaugmented feedback conditions [*F*(2, 24) = 0.048; *p* = 0.95], a highly significant Group effect was obtained in the presence of augmented feedback [*F*(2, 24) = 51.84; *p*≤0.00001]. Post-hoc tests revealed a better performance in the active compared to the passive [*p* = 0.00013] and control [*p* = 0.00013] groups. Furthermore, the passive training group showed a significantly better performance than the control group [*p* = 0.0042].

Variability. While there was no significant main effect of Group [*F*(2, 24) = 2.22; *p* = 0.13], the main effect of Feedback reached significance [*F*(1, 24) = 38.95; *p*≤0.000001], as well as the Group×Feedback interaction [*F*(2, 24) = 8.79; *p* = 0.001] (see Figure 4). Separate ANOVAs for each feedback condition were performed. When augmented feedback was not present, all groups performed equally [*F*(2, 24) = 1.00; *p* = 0.38]. The effect of group was highly significant when feedback was provided [*F*(2, 24) = 12.12; *p* = 0.00023], i.e., the active training group outperformed the control and the passive groups [*p* = 0.00029; *p* = 0.0080, respectively].

#### Performance of the bimanual movement pattern: 1∶2 frequency ratio

Accuracy. Only the main effect of Feedback (see Figure 5) was significant [*F*(1, 24) = 5.37; *p* = 0.029]: the mean score during the feedback versus no feedback condition was 0.23 vs. 0.41, respectively. The post-hoc tests revealed that accuracy was higher when feedback was present [*p* = 0.034]. The effect of Group was not significant [*F*(2, 24) = 1.82; *p* = 0.18], nor was the Group×Feedback interaction [*F*(2, 24) = 0.46; *p* = 0.64].

Variability. Whereas the main effect of Group was significant [*F*(2, 24) = 6.55; *p* = 0.0054] (active: *M* = 0.19; passive: *M* = 0.22; control: *M* = 0.33), neither the main effect of Feedback [*F*(1, 24) = 2.64; *p* = 0.12], nor the Group×Feedback interaction reached significance [*F*(2, 24) = 1.15; *p* = 0.33]. The post-hoc tests on Group revealed that both the active and passive training groups were superior to the control group [*p* = 0.0082; *p* = 0.024, respectively] whereas the active and passive groups did not differ from each other [*p* = 0.85].

#### Percentage improvement across measures

Here, we aimed to directly compare the improvement on two metrics of the task, namely, the relative phase relation (RPH) and the cycling frequency ratio (CFR) of 1∶2. Inspection of the performance curves led us to hypothesize that most improvement was gained for CFR. To obtain comparable measures, we standardized the improvements between pre-test and retention by transformation to percentages. Here we only considered feedback trials, as no improvements toward retention were observed under no feedback conditions for RPH measures.

Accuracy. A 3×2 ANOVA was conducted with factors Group×Type of Metric (RPH or CFR). The main effects of Group [*F*(2, 24) = 7.55; *p* = 0.0029] and Type of Metric [*F*(1, 24) = 27.98; *p* = 0.00002] were significant. There was a significant Type of Metric×Group interaction [*F*(2, 24) = 3.59; *p* = 0.043], suggesting that improvement on CFR and RPH was different between training groups. Post-hoc tests indicated that improvement scores on both CFR and RPH were high and comparable in the active training group [*p* = 0.91]. However, the passive training and the control group showed a stronger improvement on CFR than on RPH [*p* = 0.034; *p* = 0.00049, respectively]. This suggests that the absence of active practice affected RPH much stronger than CFR.

Variability. The 3×2 ANOVA revealed main effects of Group [*F*(2, 24) = 4.39; *p* = 0.024] and Type of Metric [*F*(1, 24) = 9.40; *p* = 0.0053]. Most improvement was obtained in RPH (RPH: *M* = 37.11; CFR: *M* = −14.77) [*p* = 0.0050]. Improvement neither differed between groups on RPH, nor on CFR [all *p*>0.2; all *p*>0.1, respectively]. Type of Metric and Group did not interact [*F*(2, 24) = 0.066; *p* = 0.94].

## Discussion

We investigated whether provision of prescriptive proprioceptive information by means of passive movement training supports learning of a new bimanual coordination mode and whether these effects were comparable to those obtained with active training. This question was addressed with respect to the global timing (frequency ratio) as well as the spatiotemporal (relative phase) relationship between the limbs. In principle, a 1∶2 frequency ratio can be obtained with any relative phase relationship, underscoring their relative independence as performance metrics. During the acquisition phase, relative phase accuracy showed a gradual tendency to be superior in the passive as compared to the control group and this effect became significant during the retention test trials with augmented feedback. However, performance in the passive group was significantly lower than the active group. Furthermore, these effects did not carry over to conditions without augmented feedback. With respect to the 1∶2 frequency ratio, both training groups behaved more similarly and made significantly more progress than the control group during the acquisition phase, and this effect was preserved during the retention phase. The latter effect was evident under both test conditions (augmented and nonaugmented).

However, none of the groups were able to reproduce the required relative phase when augmented feedback was removed. Whereas non-intrinsic coordination modes (such as the present pattern) are usually difficult to perform, augmented visual feedback (such as Lissajous figures) can help overcome these constraints, allowing for a stable execution of even highly complex movements [2], [15], [19]. Lissajous feedback promotes the integration of both movements into a unified pattern or gestalt, also called ‘motor binding’ [37]. The powerful impact of such information sources has led to the idea that coordination constraints are perceptual and can be overcome by providing perceptual information to correct and shape movement patterns [46], [49], [50]. Nevertheless, training subjects with augmented feedback does not necessarily generalize to nonaugmented feedback conditions, reflecting vulnerability of the internal movement representation [14], [51] due to dependence on augmented feedback [22]. A neural correlate of this effect was recently obtained in a medical imaging study in which visual processing areas in the brain, tailored to augmented Lissajous feedback processing during bimanual coordination learning, remained active when the latter feedback was withheld at later test trials [17]. This suggests that the augmented feedback had become part of the brain network responsible for production of that coordination pattern.

It is important to emphasize this performance dependence on provision of augmented feedback. However, when such feedback is not provided during training, learning of new complex coordination modes may be more difficult. A potential solution is to gradually wean the performer from the augmented feedback condition to promote reliance on the naturally available feedback sources required to maintain performance in the absence of augmented feedback (for example, fading feedback) [14], [47]. Furthermore, it should be noted that the use of a metronome to pace participant's movement tempo can attenuate learning effects [51].

Our hypothesis that provision of prescriptive proprioceptive information by means of passive training can induce motor learning (i.e., that performance would be greater than control) was partially confirmed. At the retention test, significant improvement was observed with respect to the new phase relation when the passive group was compared to the control group but the effect was smaller than in the active group and only present with augmented feedback. Conversely, with respect to the frequency ratio, performance levels between the active and passive group were similar at the retention test and did carry over to nonaugmented feedback test conditions. Because this is the first study addressing the acquisition of new bimanual coordination patterns through passive training, relating these findings to other studies is difficult. In recent learning studies using a unimanual movement [28], proprioceptive experience was found to induce motor learning. The benefits of proprioceptive practice are likely due to provision of a reference of correctness that can be used to eventually guide motor output (e.g., [20], [21], [36], [52]). This proprioceptive input may be integrated with the available perceptual sources to support active movement reproduction. However, the sensory representation was apparently not sufficiently developed to support the production of the 90° phase offset in nonaugmented feedback conditions. This may be a consequence of the high complexity of the present task (see further).

The behavioral communalities between passive and active training may converge with partially similar processes at the brain level. A positron emission tomography (PET) study identified a large overlap in brain activity between passive and active movements. Their common activation patterns are likely attributed to processing of afferent input, suggesting that part of the brain activity associated with active movements is actually related to afferent processing [53]. Passive movements may thus help induce cortical reorganization for recovery in stroke patients and other patient groups, particularly when they are unable to move their limbs [54], [55]. All together, the existing studies point to communalities in brain activation patterns between passive and active movement experience and hence their (at least partially) similar impact on performance and learning. Passive training may enhance the formation of a sensory representation associated with correct movement that serves as a reference of correctness against which the performer's actual movement is compared.

Our data appear to suggest that it is easier to learn a new frequency ratio than a new phase relation. Whereas a frequency ratio refers to macroscopic global timing, relative phasing deals with spatiotemporal organization at the microscopic time scale. Our daily experience with musical rhythms is more in alignment with the macroscopic than microscopic time scales. Furthermore, the integer frequency ratio we trained was relatively simple compared to performance of noninteger ratios. Finger tapping studies have provided massive evidence for the higher difficulty and lower performance levels during production of noninteger as compared to integer rhythms [42], [44]. This may have contributed to relatively better learning of the frequency ratio than relative phasing and more optimal transfer to nonaugmented feedback conditions (i.e., in the absence of augmented visual feedback and metronome pacing). Moreover, movement complexity may also determine degree of transfer of relative phase features to nonaugmented feedback conditions. Previous studies on 1∶1 bimanual patterns with a 90° phase offset have demonstrated better (but not perfect) transfer to nonaugmented feedback conditions [2], [18] than the present study [19]. Furthermore, our previous work has shown that performing the 2∶1 frequency ratio according to an in-phase mode (resulting in a ‘C’ Lissajous configuration) can be preserved more successfully than the 90° phase offset mode in the absence of augmented feedback [19]. The 0° phase difference at the peak displacement positions of both limbs in the in-phase variant allows synchronization of the reversals in movement direction, resulting in better performance and more rapid learning. Conversely, the sequential reversals in movement direction of both limbs during the 90° task variant is more complicated [19].

Even though passive and active training were equally useful in acquiring the 1∶2 frequency ratio, the active group was more successful than the passive group in producing the required relative phasing when perceptual information in the form of a Lissajous plot was available. We hypothesize that the superiority of the active training group is a consequence of a more active involvement in error detection and correction processes, leading to movement planning and replanning processes. This discrepancy between how movements ‘should be’ and ‘have actually been’ performed is lacking in the passive training group because experience is limited to exposure to the correct sensory consequences of movement only. In other words, learning may benefit from experience with movement error and this may amplify the perception-action interplay.

Although (to the best of our knowledge) studies comparing active and passive training groups for skill acquisition are virtually absent, some indications in the literature are consistent with the present observations. Neurophysiological evidence for example, points to a deeper neural encoding of actively compared to passively performed movements as evidenced by increased corticomotor excitability evoked by TMS [41], [56]. Computational neuroscience perspectives emphasize that active movements are accompanied by efference copies, or internal models, which are required for prediction of the sensory consequences of the action and current state estimation [57], [58]. Because passive movements are not accompanied by generation of internal models, the actor cannot learn from error detection/correction to update and refine the internal model. The implication for therapeutic interventions is that when robots are used to assist in (re-) acquiring a movement skill, they should impose as little movement as possible to allow patients to move actively as much as possible. Furthermore, gradual weaning from augmented feedback is also critical for transfer to everyday contexts.

In summary, in comparing active versus passive movement training during acquisition of a new complex bimanual task, we find that both types of movement training lead to comparable learning of a frequency ratio but active training leads to superior performance of a new relative phase mode in the presence of augmented feedback. Nevertheless, our results suggest that some degree of learning is possible with prescriptive proprioceptive input, depending on the complexity of the task and the instructional context.
